# Exosomes from adipose-derived stem cells promote angiogenesis and reduce necrotic grade in hindlimb ischemia mouse models

**DOI:** 10.22038/IJBMS.2023.67936.14857

**Published:** 2023-04

**Authors:** Trinh Hoang-Nhat Nguyen, Phuc Van Pham, Ngoc Bich Vu

**Affiliations:** 1 Stem Cell Institute, University of Science Ho Chi Minh City, Viet Nam; 2 Viet Nam National University, Ho Chi Minh City, Viet Nam; 3 Laboratory of Stem Cell Research and Application, University of Science Ho Chi Minh City, Viet Nam

**Keywords:** Acute, Adipose-derived stem cells, Angiogenesis, Exosome, Extracellular vesicles, Limb ischemia

## Abstract

**Objective(s):**

Acute hindlimb ischemia is a peripheral arterial disease that severely affects the patient’s health. Injection of stem cells-derived exosomes that promote angiogenesis is a promising therapeutic strategy to increase perfusion and repair ischemic tissues. This study aimed to evaluate the efficacy of adipose stem cell-derived exosomes injection (ADSC-Exos) in treating acute mouse hindlimb ischemia.

**Materials and Methods:**

ADSC-Exos were collected via ultracentrifugation. Exosome-specific markers were analyzed via flow cytometry. The morphology of exosomes was detected by TEM. A dose of 100 ug exosomes/100 ul PBS was locally injected into acute mice ischemic hindlimb. The treatment efficacy was evaluated based on the oxygen saturation level, limb function, new blood vessel formation, muscle structure recovery, and limb necrosis grade.

**Results:**

ADSC-exosomes expressed high positivity for markers CD9 (76.0%), CD63 (91.2%), and CD81 (99.6%), and have a cup shape. After being injected into the muscle, in the treatment group, many small and short blood vessels formed around the first ligation and grew down toward the second ligation. The SpO2 level, reperfusion, and recovery of the limb function are more positively improved in the treatment group. On day 28, the muscle’s histological structure in the treatment group is similar to normal tissue. Approximately 33.33% of the mice had grade I and II lesions and there were no grade III and IV observed in the treatment group. Meanwhile, in the placebo group, 60% had grade I to IV lesions.

**Conclusion:**

ADSC-Exos showed the ability to stimulate angiogenesis and significantly reduce the rate of limb necrosis.

## Introduction

Acute limb ischemia (ALI) is defined as a condition in which limb perfusion is suddenly decreased, causing a potential threat to the surviviAdipose-derivedects 1.5 patients per 10,000 people each year. ALI is common in the lower extremities in 9 to 16 new patients and the upper extremities in 1 to 3 new patients per 100,000 individuals per year (1, 2). This disease can be caused by a variety of conditions, such as trauma (5%), arterial embolism (30%), and thrombosis-related conditions (65%) (3). Its main clinical manifestation occurs within 2 weeks after the beginning of symptoms (4). The symptoms of ALI develop within several minutes to hr or days. They range from new or intermittent claudication to pain at rest, paranesthesia, muscle weakness, and paralysis. In severe cases, limb gangrene may lead to limb amputation or death (1). Moreover, patients with ALI are at high risk for complications, including pulmonary problems, renal function worsening, myocardial infarction, and heart failure (5). Within 30 days after presentation, 15-25 % of patients with ALI die from associated diseases (4).

Revascularization to restore blood flow is the primary aim of the current treatment options (1). The majority of patients with ALI require endovascular intervention (cryoplasty or implantation of stent grafts or drug-eluting balloons or stents), bypass surgery, or plasminogen activator medication (1, 4). Despite all medical advances, 20-45% of patients are not eligible for surgery or experience treatment failure. This subgroup of patients can experience serious complications, such as amputation and even death (6, 7). Therefore, novel treatment methods to improve limb perfusion among patients with ALI, especially those considered to have “no treatment option,” are imperative. Targeting microvascular regeneration using therapeutic stem cells is a potential strategy for patients with ALI (8).

In recent years, many studies have applied different stem cell sources, such as bone marrow-derived stem cells, adipose-derived stem cells (ADSCs) (9), menstrual blood cells (10), and placenta-derived mesenchymal stem cells (MSCs), in the treatment of ALI (11). Stem cell therapy has shown beneficial results in the treatment of this disease. As the scientific basis underlying the effectiveness of stem cell therapy, extracellular secretion from stem cells is evaluated as the main mechanism for regenerating ischemic injury. ADSCs have been proven to be superior in the secretion of bioactive factors related to angiogenesis and tissue regeneration (12).

Accumulating evidence supports that extracellular vesicles (EVs), especially exosomes, mediate the regenerative capacity of MSCs (13, 14). EVs are nanosized cell-derived vesicles that exist in most body fluids, such as blood and urine, and cell culture media. Exosomes, microvesicles, and apoptotic bodies are the three primary subtypes of EVs (15). Exosomes are 30–100 nm in diameter and derived from the budding of multivesicular bodies (16). Exosomes derived from MSCs contain a variety of cargo categories, including bioactive lipids, functional proteins, and RNAs (17, 18). Moreover, EVs and exosomes have the capacity to transfer bioactive factors and regulate tissue regeneration by reprogramming recipient cells (19, 20).

Exosomes can promote angiogenesis by transporting proangiogenic miRs from MSCs, such as miR-125a (21), miR-126 (22), and miR-30b (20), to endothelial cells and by repressing DLL4. In a previous study, miR532-5p, miR148a, let-7f, and miR378 were enriched in MSC-EVs and regulated many biological processes, including angiogenesis, apoptosis, proteolysis, and transcription, in recipient cells (23). *In vitro*, endothelial cells treated with MSC-exosomes increased the expression of molecules involved in angiogenesis, such as angiogenin, HIF-1a, VEGFA, VEGFR-2, ANG-1, PDGFA, PGF, bFGF, TGFB1, bFGFR, and IL-8 (24, 25). Exosomes derived from MSCs were used to treat many diseases, while those derived from EPCs, iMSCs, and placenta-derived MSCs enhanced the density of microvessels and blood perfusion in mouse models of ischemia (19, 25, 26). Umbilical cord MSC-exosomes promoted angiogenesis in a model of cutaneous burns by activating Wnt/β-catenin (27). Generally, MSC-exosomes contain cytokines and growth factors related to muscle repairs, such as VEGF, IL-6, FGF-2, GCSF, and PDGF-BB, which encourage muscle repair by promoting angiogenesis and myogenesis (24).

Therefore, the purpose of this research was to assess the treatment efficacy of exosomes from ADSCs (ADSC-Exos) in mouse models of acute hindlimb ischemia.

## Materials and Methods

***Characteristics of the ADSCs***

Frozen human ADSCs were provided by SCI Biobank (Stem Cell Institute, HCMC, VN). Cryovials were thawed in a thermostatic bath at 37 ^°^C. The cell cryopreservation medium was removed via centrifugation at 1,500 rpm for 5 min. The cells were cultured in MSCCult I medium (Regenmedlab, VN) at 37 ^°^C and 5% CO_2_. The medium was refreshed every 2-3 days depending on the proliferation of the cells until they reached the desired density.

Three characteristics of the designated MSCs, including the expression of MSC markers, ability to differentiate into functional cells, and the ability to adhere to plastic surfaces, were then evaluated. First, 10,000 cells were stained with antibodies against CD14 FITC, CD34 FITC, CD19 PerCD, CD45 APC, CD90 PE, CD73 PE, CD105 PerCD, and HLA-DR FITC (Miltenyi Biotec, Germany) for 15 min in the dark. The antibodies were washed two times using phosphate buffer solution (PBS) via centrifugation at 3,500 rpm for 5 min. The cells were resuspended in 300 µl of PBS and analyzed using the BD FACSCalibur system. Second, the ADSC candidates were cultured in StemPro^®^ Adipogenesis Differentiation Media, StemPro^®^ Osteogenesis Differentiation Media, and StemPro^®^ Chondrogenesis Differentiation Media (Gibco/Thermo Fisher Scientific, MA, USA) to detect their ability to differentiate into adipocytes, osteoblasts, and chondroblasts, respectively. On day (D) 14, the cells were stained with Oil Red O (Sigma-Aldrich, MO) to observe the appearance of the adipocytes. On D21, the cells were stained with Alizarin Red (Sigma-Aldrich, MO) to determine the accumulation of Ca^2+^ and Mg^2+^ in the osteoblasts as well as with Alcian Blue (Sigma-Aldrich, MO) to determine the synthesis and storage of proteoglycans in the chondroblasts. Finally, the ADSC candidates were observed under a microscope to check the cell adherence on the plastic surface.

***Collection and identification of the stem cells-derived exosomes***

The human ADSCs from pass 3 to pass 7 were cultured in MSCCult I medium to collect the supernatant. The supernatant was centrifuged at 300×g for 10 min to remove the remaining cells. Extracellular secretion of the human ADSCs was induced via ultracentrifugation at 4 ^°^C (28). Briefly, the supernatant was transferred to a new centrifuge tube and further centrifuged at 2,000 × g for 20 min to remove dead cells and large debris. Thereafter, the supernatant was transferred to a new ultracentrifuge tube and centrifuged at 10,000×g for 30 min to remove small debris and large bags. The collected fluid was then transferred to a new ultracentrifuge tube and ultracentrifuged at 100,000×g for 70 min. After this step, the supernatant was removed, leaving approximately 2 mm of the supernatant above the pellet. The pellet was redissolved in PBS and ultracentrifuged at 100,000 × g for 70 min. The pellet was kept in 100 μl of PBS. The ADSC-Exos candidates were then stored at -80 ^°^C for further experiments.

The Bradford assay was used to evaluate the total protein concentration of the ADSC-Exos (28). The BSA standards and ADSC-Exos were thawed at 4 ^°^C. In a flat-bottomed 96-well plate, 10 µl of each PBS was loaded into a blank well. Thereafter, 10 µl of each BSA standard (Thermo Fisher Scientific, MA) was added to the wells. Five microliters of PBS were loaded in the well containing the ADSC-Exos sample, and 5 µl of the sample was then added to each well. Each solution was repeated in three wells, and 300 µl of Coomassie blue solution was then added to each well. The OD at 595 nm was read within 10 min.

The ADSC-Exos were identified based on the expression of specific markers, including CD63, CD9, and CD81, and via flow cytometry (28). Briefly, 50 µg of the ADSC-Exos was incubated with 1 µl of latex aldehyde/sulfate beads for 15 min at room temperature (RT). PBS was then added to a final volume of 1 ml and incubated overnight at 4 ^°^C. Thereafter, 110 µl of 1 M glycine was added to the abovementioned solution, mixed gently, and incubated for 30 min, followed by centrifugation at 4,000 rpm at RT for 3 min. In the next step, the bead pellet was resuspended in 1 ml PBS/0.5% BSA and centrifuged at 4,000 rpm for 3 min to discard the supernatant. The bead pellet was resuspended in 0.7 ml PBS/0.5% BSA and divided into 2 ml tubes. One microliter of antibody was added to the tube and incubated for 30 min at 4 ^°^C in the dark. Next, the tubes were centrifuged at 4,000 rpm for 3 min to remove the supernatant. Finally, the bead pellet was suspended in 300 µl of PBS and analyzed on a BD FACSMelody cell sorter (BD BioSciences, NJ).

***Mouse model of acute hindlimb ischemia***

All animal experiments were approved by the *Institutional Animal *Care and Use* Committee*, Stem Cell Institute (HCMC, VN). Mice over 6 months old were used to create the acute ischemic hindlimb model according to the protocols published by Vu *et al*. (29). Briefly, the mice were anesthetized via intramuscular injection of 4 mg/kg xylazine (VIME-LAZIN, Vemedim, Vietnam) and 5 mg/kg Zoletil (Virbac, France). Hairy thighs were shaved and disinfected with a 10% povidone-iodine solution (Pharmedic, Vietnam). An incision approximately 1.5 cm long was created in the skin. The femoral artery and vein near the abdomen were separated from the muscle and nerves and ligated at two locations-above and below the superficial caudal epigastric artery. Next, the two ligated blood vessels were cut using scissors. Finally, the skin was stitched, and the wound area was covered in a povidone-iodine solution.

***ADSC-Exos injection in the acute hindlimb ischemic mice***

The acute hindlimb ischemic mice were divided into two groups. The placebo group was injected with 150 µl PBS, while the treatment group was injected with 100 µg ADSC-Exos suspended in 150 µl PBS. ADSC-Exos or PBS was injected directly into the muscle at the burn sites immediately after the models were established. All mice were followed up for 28 days after the injection.

***Evaluation of limb recovery after ADSC-Exos injection***

The severity of the ischemic injury was evaluated based on the grade of limb necrosis according to the guidelines by Goto *et al*. (28): grade 0, normal limb without necrosis; grade I, black toenails with necrosis limited to the toes; grade II, necrosis extending to the foot; grade III, necrosis extending to the knee; and grade IV, necrosis extending to the hip or loss of the entire hindlimb.

The improvement in perfusion was assessed using the trypan blue flow assay. One hundred microliters of 0.4% trypan blue solution were injected into the tail vein of the mice. The time to staining of the toes and footpad was recorded on D3, D7, D14, and D28 and compared with that in normal mice.

Blood flow up to the limb was indirectly evaluated based on the blood oxygen level. The peripheral oxygen saturation (SpO_2_) level was monitored using Contec08A+ SpO_2_ Probe on D3, D7, D14, D21, and D28 and compared with that in normal mice.

Limb function recovery was assessed by observing the movements of the mouse limbs while moving on a plane and by counting the pedal frequency of the hindlimbs over a 10-sec period.

The changes in tissue structure were analyzed through histological experiments on D3 and D28 after injection. The mice were euthanized, and the hindlimb muscle between the two ligated blood vessels was biopsied and fixed in a 4% paraformaldehyde solution overnight. Thereafter, the tissue samples were cut into 3-µm sections and stained with hematoxylin and eosin (H&E) at Cho Ray Hospital (Ho Chi Minh City, Vietnam). All slides were observed using an inverted microscope (Carl Zeiss, Germany).

Neovascularization was assessed on D7 and D28 after surgery. The mice were euthanized, and the formation of new blood vessels was observed via stereoscopic microscopy (Carl Zeiss).

***Statistical analysis***

Data were presented as means±standard deviations. All statistical analyses were performed using GraphPad Prism 8.0. Differences were considered significant at *P*<0.05.

## Results

***Characterization of the ADSCs***

**Figure 1:**
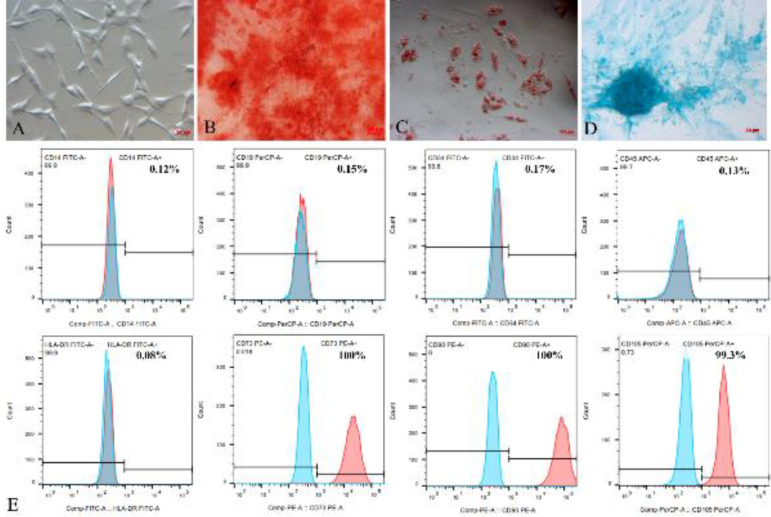
Human adipose-derived stem cells expressed the MSC phenotype proposed by ISCT

After thawing, the human ADSCs were reconfirmed to be MSCs. The ADSCs were able to adhere and spread on the culture vessel surface and exhibited classic fibroblast-like cell morphology (Figure 1A). After 21 days of culture in the osteogenic induction medium, extracellular matrix Ca^2+^ accumulation was observed (Figure 1B). The formation of lipid droplets in the cells was observed after 7-14 days of culture in the adipogenic induction medium. The lipid droplets were confirmed based on positive staining with Oil Red O (Figure 1C). After chondrogenic induction, the cells aggregated and increased the production of the extracellular matrix. The induced cells were positive for Alcian Blue staining 21 days after induction (Figure 1D). These results showed that the ADSC candidates were able to differentiate into functional mesoderm cells. On flow cytometry, the cells expressed MSC-specific markers. Specifically, these cells were negative for CD14 (0.12%), CD19 (0.15%), CD34 (0.17%), CD45 (0.13%), and HLA-DR (0.08%) surface expression but were positive for CD73 (100%), CD90 (100%), and CD105 (99.3%) surface expression (Figure 1E). Thus, according to the standards of MSCs of the International Society for Cell and Gene Therapy (29), the obtained human ADSCs were confirmed to be MSCs.

***Characterization of the human ADSC-Exos***

**Figure 2:**
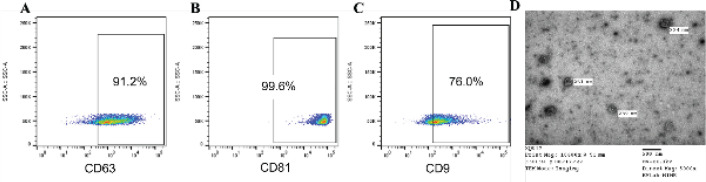
Flow cytometry characteristics of isolated exosomes from human adipose-derived stem cells. They expressed markers of CD63 (A), CD81 (B), and CD9 (C), and have a morphology like disk or cup (D)

The protein concentration of the ADSC-Exos per milliliter was 1.42±0.2 µg. Flow cytometry showed that the isolated ADSC-Exos expressed all three markers (CD9, CD63, and CD81). Among the three analyzed markers, CD81 had the highest expression, while CD9 had the lowest expression. The percentages of exosomes positive for CD63 (Figure 2A), CD81 (Figure 2B), and CD9 (Figure 2C) were 91.2%, 99.6%, and 76.0%, respectively. ADSC-Exos also showed the morphology like a disk or cup (Figure 2D).

***Recovery of the peripheral capillary SpO***_2_ ***level after ADSC-Exos injection***

**Figure 3:**
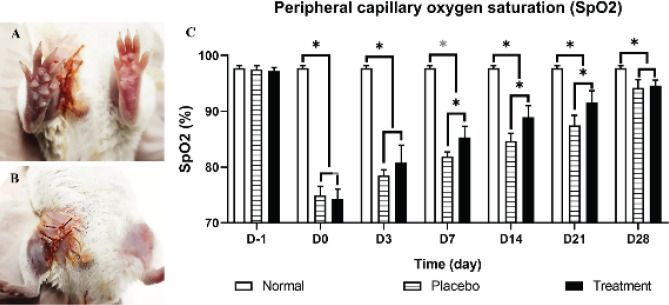
Recovery of the peripheral capillary SpO2 level after ADSC-Exos injection

Two to three hr after acute disturbance of blood flow, signs of peripheral cyanosis in the limb were observed in both groups (Figures 3A and B). The SpO_2_ level recovered more rapidly in the treatment group than in the placebo group. The average SpO_2_ level in the normal mice was 97.73%±0.46%. After 3 hr of ischemia, the SpO_2_ levels in the placebo and treatment groups were 74.93%±1.61% and 74.33%±1.74%, respectively. The level in both groups significantly decreased compared with that in the normal mice (*P*<0.05). From D7 to D21, the SpO_2_ level in the treatment group increased from 85.31%±1.97% to 91.53%±2.16% (*P*<0.05), while that in the placebo group increased from 81.91%±1.15% to 87.49%±1.77% (*P*<0.05). On D28 after injection, the SpO_2_ level in both groups was over 94%, and there was no significant difference found (Figure 3C).

***Recovery of the blood flow to the feet***

**Figure 4:**
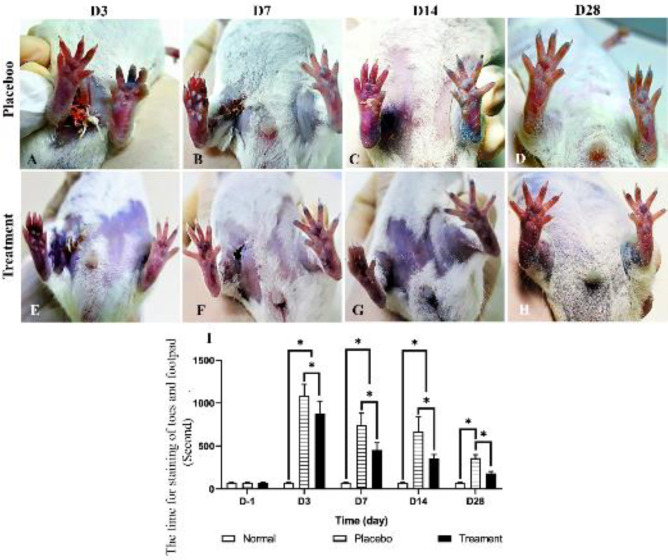
Recovery of the blood flow to the feet

In the normal mice, the paw pads and toes in both hindlimbs simultaneously appeared blue 71.00±1.87 sec after trypan blue injection into the tail vein (Figure 4). On D3, the average time to blue staining of the ischemic hindlimbs was 1089±131.95 sec in the placebo group and 880.40±142.27 sec in the treatment group (*P*<0.05). The average time to trypan blue staining of the ischemic hindlimbs in the treatment group decreased considerably from D7 to D28. On D28, the paw pads and toes stained blue after 181.90±22.42 sec in the treatment group. The time did not significantly differ between the treatment group and normal mice (*P*<0.05). However, it significantly differed between the treatment and placebo groups (*P*<0.05). The restoration of blood flow was evaluated using the trypan blue assay in the mice, which revealed that the blood flow in the treatment group was more improved than that in the placebo group.

***Recovery of the limb function***

**Figure 5:**
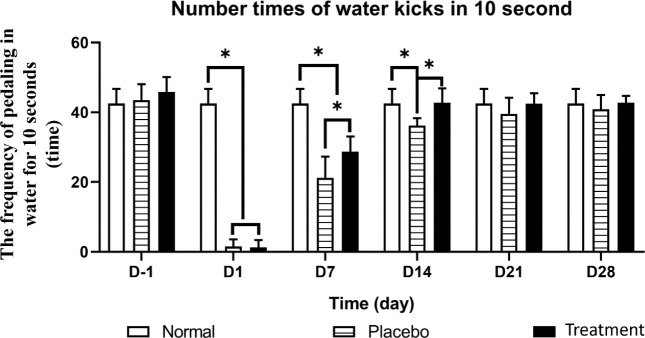
Recovery of the limb function. The mobility of mice climbs sharply decreased immediately after induced ischemia in both groups. The treatment group completely recovered after 14 days, while the untreated group took up to 21 days

Three hours after surgery, the mobility of the ischemic hindlimbs in both groups was dramatically reduced, and the hindlimbs were dragged when the mouse moved. The mice with normal limb function were used as a reference to evaluate mobility. In the normal mice, the pedal frequency was 45.03±4.45 times/10 sec; in the two groups, almost all ischemic hindlimbs lost mobility in water after 1 day. In the treatment group, the hindlimb function was restored to normal after 14 days (42.8±4.07 times/10 sec), and there was no significant difference from the normal mice (*P*<0.05). Meanwhile, in the placebo group, it took up to 21 days for the limb function to return to normal (39.53±4.64 times/10 sec) (Figure 5).

***Neovascularization***

**Figure 6:**
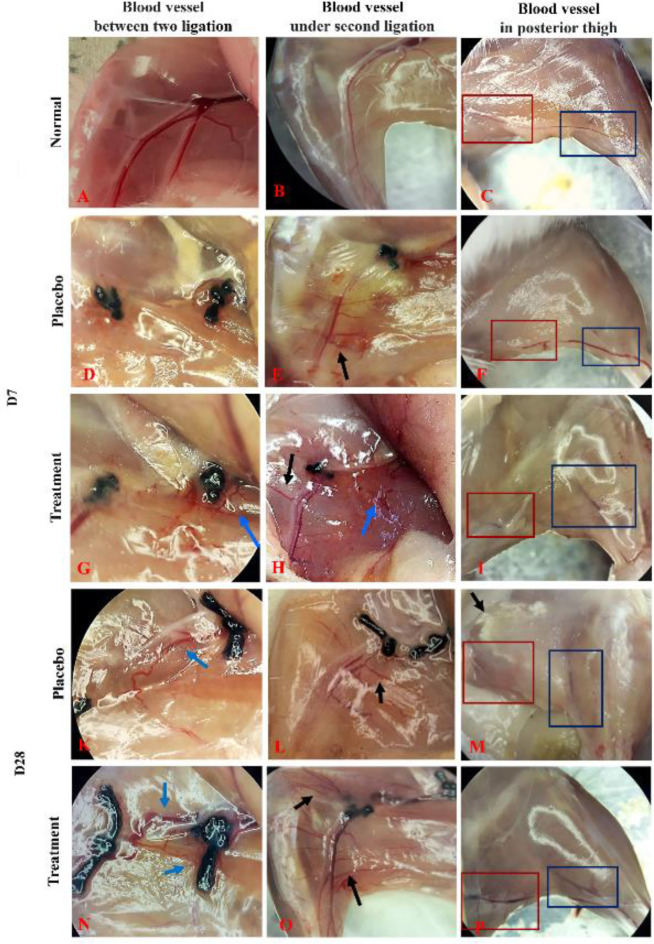
Formation of new blood vessels. Vascular structure in normal mice (A-C). After 7 days, in comparison to normal mice, the blood vessel under second ligation developed in both the placebo group (D-F) and the treatment group (G-H). After 28 days, the development of new blood vessels connected between the two ligation sites in both the untreated group (K-M) and treated mice (N-M) was stronger. However, the vascular development under second ligation and posterior thigh in treated mice were also stronger than in untreated mice groups. The arrows point to new blood vessels

Stereoscopic microscopy showed that neovascularization improved in both groups. Vascular growth was stronger in the treatment group than in the placebo group. After 7 days, blood vessels under the second ligation in both groups developed in response to ischemia compared with those in the normal mice. Multiple vascular branches arising from the saphenous artery developed laterally (Figures 6B2 and B3, black arrow). Remarkably, many small and short blood vessels formed around the first ligation and grew down toward the second ligation in the treatment group (Figure 6A3, blue arrow); however, these were faintly expressed in the placebo group. After 28 days, the new vasculature developed wavy tortuous shapes connecting the two ligations. Vascular development under the second ligation and posterior thigh was also stronger in the treatment group than in the placebo group.

***Histological structure of the muscle tissue***

**Figure 7:**
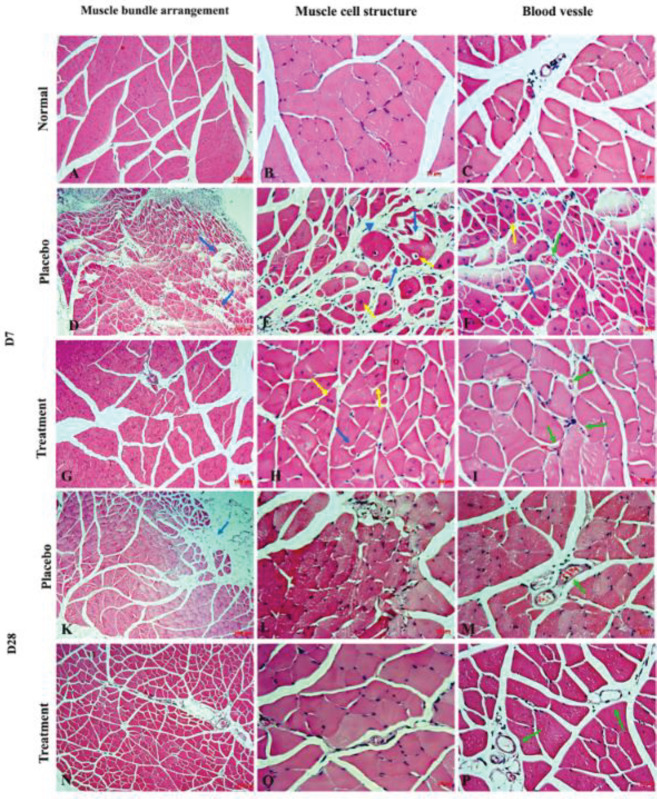
Histological muscle tissue structure at 7 days and 28 days after injection

Muscle tissue structure restoration and blood vessel formation were assessed via histology. H&E staining showed that the skeletal muscle of the normal mice had an orderly arrangement of muscle cells in bundles. In cross-section, the muscle cells were polygonal in shape and relatively uniform in size. Each muscle cell had multiple nuclei distributed peripherally and stained blue‒black with hematoxylin. The cytoplasm was stained pink with eosin. However, the histological muscle structure of the treatment and placebo groups exhibited microscopic changes related to degeneration at 7 days after ischemic induction. In the placebo group, the tissue structures were destroyed and lost muscle bundles (Figure 7A2, blue arrow). The muscle cells were disorderly arranged and expressed abnormal shapes (Figure 7B2, blue head arrow). The cytoplasm exhibited fragmentation and shrinkage (Figure 7B2, blue arrow), while the nuclei were concentrated near or in the cytoplasm (Figure 7B2, yellow arrow). The muscle lesions were milder in the treatment group than in the placebo group. In the treatment group, the cells retained their normal shape and were arranged in an orderly manner (Figure 7B3). The small blood vessels were also observed to be interspersed between the muscle cells. After 28 days, the muscle structure of the mice without hindlimb loss in both groups significantly improved. However, the muscle structure at the postinjury sites appeared in tissue areas without recovery in the placebo group (Figure 7A4, blue arrow). In contrast, the muscle structures were similar to normal tissue structures in the treatment group (Figure 7A5). The density of the blood vessels was also higher in the treatment group than in the placebo group and normal mice (Figure 7C4 and C5, green arrow).

***Reduction of the hindlimb necrosis grade***

**Figure 8:**
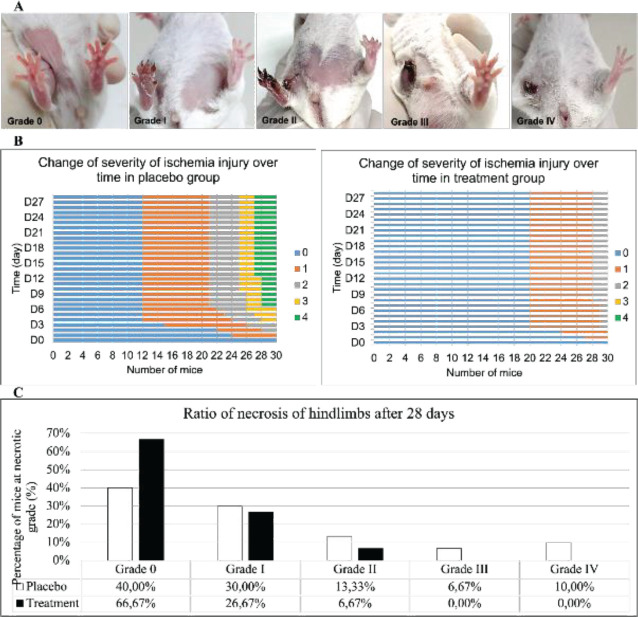
Ratio of hindlimb necrosis caused by acute ischemia in both groups. The degree of limb necrosis was assessed and categorized according to the recommendations of Goto *et al*. [28] (A). The damage process of untreated mice lasted longer than treated mice (B). The data revealed that injection of AD-EXOs dramatically reduced hindlimb necrosis compared with a placebo group (C)

The effect of the ADSC-Exos on the recuperation of acute ischemic injury in both groups was observed for 28 days. The limb necrosis grade was divided according to the classification described by Goto *et al*. (28) (Figure 8A). The ADSC-Exos considerably reduced hindlimb necrosis after ischemic injury. In the placebo group, the rate of high-grade limb necrosis rapidly increased until 13 days after ischemia induction. Meanwhile, in the treatment group, the change in the limb necrosis grade stopped on D7 (Figure 8B). In the placebo group, 40.00% of the mice fully recovered, and 60.00% had hindlimb necrosis, mainly grade I and II. The percentages of grade I, II, III, and IV necroses were 30.00%, 13.33%, 6.66%, and 10.00%, respectively. Meanwhile, in the treatment group, up to 66.67% of the mice completely recovered. Remarkably, the treatment group did not have grade III or IV necrosis, although 26.67% and 6.67% of the mice had grade I and II necroses, respectively (Figure 8C).

## Discussion

In mammals, the tetraspanin family of proteins includes more than 30 members. Tetraspanins have been found on the plasma membrane or in the endothelial compartments or lysosomes of most cell types (30). This family consists of distinct proteins that are characterized by their common specific molecular structure. Owing to the high content of tetraspanins, such as CD9, CD81, CD63, and CD82, in exosomes, they are commonly used as exosome recognition markers.

Exosomes are essential paracrine components produced by ADSCs that have various biological functions (31). In our research, exosomes were isolated from human ADSCs, and the expression of endosome-specific tetraspanins was evaluated. The analysis showed that the ADSC-Exos expressed CD9, CD63, and CD81, which is consistent with previous findings (32). However, researchers reported that the percentages of ADSC-Exos markers positive for CD9, CD63, and CD81 were 89.8%, 52.15%, and 81.18%, respectively, which were higher than those in our research (33). According to the extensive proteomic analysis of exosomes conducted by Jankovičová *et al*., exosomes derived from different cell types and even the same cells can have significant differences in their specific tetraspanin profile both qualitatively and quantitatively (34). As another example of alteration in the expression levels of these markers, ADSC-Exos isolated in the study by Mitchell expressed only CD63 without the presence of CD9 and CD81 (35). Moreover, based on the findings of the coexpression of tetraspanins, the exosomes isolated in this study included many subpopulations, which is similar to a previous report (36). For example, exosome populations either coexpressed all three markers or expressed only CD81.

In ischemic mice, the hindlimbs show significant signs of acute ischemia. The hindlimbs of mice are perfused by the external iliac artery. This artery gives rise to branches that further divide into accessory vessels that enter the muscles (37). Broken femoral blood vessels disrupt the perfusion of peripheral tissues, leading to a lack of nutrients and oxygen supply to the tissues. In ischemic tissues, the oxygen supply is significantly reduced, causing peripheral cyanosis. Peripheral cyanosis, a condition in which peripheral tissue color turns blue-violet or purple-black, is a sign of significant hypoxia. The clinical presentation of cyanosis usually occurs at an SpO_2_ level of ≤85% (37). During the treatment period, the SpO_2_ level of our treatment group increased significantly and recovered to above 85% from D7 after exosome injection, while this phenomenon occurred from D14 in the placebo group. These results show that the ability of ADSC-Exos to improve hypoxia is better than that of PBS.

The time to trypan blue staining in the paw pad also confirmed the effect of the ADSC-Exos on the blood circulation of the hindlimb ischemic mice with grade I and 0 necroses. Meanwhile, the time for blood circulation to the extremities was much shorter in the treatment group than in the placebo group.

Along with the significant physiological improvements, limb function was also more effectively restored in the treatment group. In this group, the water pedal frequency in 10 sec increased significantly and recovered to normal from D14 after transplantation, while in the placebo group, this phenomenon took 21 days to occur. Our results are consistent with previous reports. In one study, treatment with EPC-derived exosomes improved the blood oxygen concentration and shortened the recirculation time compared with PBS injection (133). Meanwhile, Hu *et al*. reported that iMSC exosome-treated anemic mice showed a restored perfusion level on D14 and had motility better than that of PBS-injected mice (25). The time to trypan blue staining in the paw pad of the mice also confirmed the efficacy of extracellular secretion in restoring blood circulation in mice with mild injury (acute I and 0). The time for blood circulation to the extremities was much shorter in our treatment group than in our placebo group.

Collateral circulation is a network of blood vessels found in most tissues. These blood vessels connect adjacent arteries, thereby limiting tissue damage caused by sudden ischemia. Collateral circulation plays an important role in maintaining blood flow and limiting damage to ischemic tissue (38). After 7 days of ischemia, the majority of the mice in both groups in our study developed collateral circulation in the lower thigh. The branch vessels tended to develop bilaterally. However, in the treatment group, small vascular structures formed from the superior knot site, which was not observed in the placebo group. The histological structure also showed that the blood vessel density in the treatment group was higher than that in the placebo group. However, the new blood vessels between the muscle fibers were small, so the blood flow to the lower extremities was very slow compared with that before vascular ablation.

In addition, the muscle tissue showed less damage and inflammation in the treatment group than in the placebo group. For up to 28 days, regeneration of the new vessels and muscles was stronger in the treatment group than in the placebo group. The vascular density was higher, and the tissue structure was more similar to normal in the treatment group than in the placebo group. As previously reported, the capillaries in mice treated with exosomes had a higher density than those in untreated mice, which was evident on D7 after transplantation (25). These results suggest that treatment with ADSC-Exos can stimulate angiogenesis and repair acute ischemic muscle injuries.

Neovascularization therapy is an effective therapeutic strategy for blood flow recovery after arterial occlusion (37, 39). The primary mechanisms are arteriogenesis and angiogenesis (39). The formation of new blood vessels as well as the development of preexisting collateral vessels could be an underlying mechanism for the improvement of blood delivery to peripheral tissues, thereby increasing oxygen supply to these tissues. Herein, the ADSC-Exos not only restored perfusion and SpO_2_ but also promoted muscle tissue structure regeneration based on the histological findings. Previous studies have also reported that exosomes derived from ADSCs could enhance the growth of skeletal muscle (40). Accordingly, these improvements led to significant decreases in limb necrosis and recovery of limb function in mice treated with ADSC-Exos.

Many studies have been conducted and have shown the important role of exocrine factors from MSCs in the regeneration of damaged tissue. These factors are capable of stimulating endogenous repair, regulating inflammation, and protecting tissues at risk (41). In culture, ADSCs contain components involved in these processes, especially signaling factors that help regenerate blood vessels and repair tissue after injury as exosomes (42). Exosomes obtained from ADSC culture have potent activities in ischemic models (43). Exosomes are highly valued for their effectiveness in the treatment of lesions. They participate in the regeneration process through the transport of biomolecules from cells to target cells in the injured tissue. These molecules activate different signaling pathways that lead to alterations in biological activities in recipient cells (19). Exosomes also show potential applications in the treatment of various pathologies: MSC-EVs promote angiogenesis in ischemic injury (44), restore poststroke function by promoting endothelial cell proliferation and capillary network expansion (45), promote collagen synthesis and angiogenesis (27), inhibit fibrosis and inflammation in the heart, and improve cardiac function in a myocardial infarction rat model (46).

The results of this study demonstrate that ADSC-Exos have the ability to significantly improve ischemia of the hindlimbs in a mouse model. ADSC-Exos promote rapid and powerful vascular regeneration, causing blood flow to circulate to the tissue injury due to ischemia. This reperfusion supports the restoration of peripheral SpO_2_ and regenerates muscle tissue, resulting in the rehabilitation of limb function and reduction in the rate of limb necrosis.

The ADSC candidates displayed the fibroblast shape (A). The cells were able to differentiate into functional cells, such as osteoblasts, adipocytes, and chondrocytes, which were confirmed by positive staining with Alizarin Red (B), Oil Red O (C), and Alcian Blue (D). The cells were strongly positive (over 98%) for markers CD73, CD90, and CD105 and did not express (less than 2%) markers CD14, CD19, CD34, CD45, and HLA-DR (E)

After 3 hr of induced ischemia, the mice’s hindlimbs expressed cyanosis at the foot and thighs caused by reduced oxygen saturation (A, B). During 28 days of monitoring, the SpO_2_ change in both groups was recorded to show that the recovery of SpO_2_ in the treatment group was better than in the placebo group (C)

On day 3 (A, E), the time ischemic hindlimb to stain with trypan blue was longer than in normal mice in both placebo (A-D) and treatment (E-H) groups. From days 7 (B, F), 14 (C, G), and 28 (D, H), the average time for ischemic hindlimbs stained with trypan blue in the treatment group treated with ADSCs-EXOs decreased more significantly than in the placebo group (I)

The histological structure of muscle tissue in normal mice showed polygonal-shaped muscle cells orderly arranged in muscle bundles (A-C). After 7 days, muscle structure in both the placebo group (D-F) and the treatment group (G-I) expressed degeneration. After 28 days, the muscle structure of mice without loss of hindlimb in untreated mice (K-M) and treated mice (N-P) was dramatically improved. Furthermore, blood vessel denseness in the treatment group (P) was higher than in the placebo group (M). The green arrows point to blood vessels

## Conclusion

Exosomes derived from human ADSCs significantly improve acute ischemic symptoms in a mouse model. ADSC-Exos accelerate the recovery of physiological states, such as peripheral SpO_2_, blood circulation, and limb motion function. Injection of ADSC-Exos enhances vascular remodeling and repair of damaged muscle tissue, thereby reducing the limb necrosis grade in treated mice compared with that in untreated mice. ADSC-Exos are confirmed to be a potential treatment for hindlimb ischemic diseases.

## Authors’ Contributions

THNN, PVP, and NBV designed the experiments; THNN and NBV performed experiments and collected data; THNN, PVP, and NBV discussed the results and strategy; NBV supervised, directed, and managed the study; THNN, PVP, and NBV approved the final version to be published.

## Conflicts of Interest

The authors declare that they have no competing interests. 
